# Intracranial hypertension after cerebral venous thrombosis—Risk factors and outcomes

**DOI:** 10.1111/cns.14194

**Published:** 2023-03-28

**Authors:** Huimin Wei, Huimin Jiang, Yifan Zhou, Lu Liu, Chen Zhou, Xunming Ji

**Affiliations:** ^1^ Beijing Advanced Innovation Center for Big Data‐Based Precision Medicine, School of Biological Science and Medical Engineering Beihang University Beijing China; ^2^ Laboratory of Brain Disorders, Ministry of Science and Technology, Collaborative Innovation Center for Brain Disorders, Beijing Institute of Brain Disorders Beijing Advanced Innovation Center for Big Data‐based Precision Medicine, Capital Medical University Beijing China; ^3^ Department of Neurology, Xuanwu Hospital Capital Medical University Beijing China; ^4^ Department of Neurosurgery, Xuanwu Hospital Capital Medical University Beijing China

**Keywords:** cerebral venous thrombosis, cerebrovascular disease, intracranial hypertension, raised intracranial pressure, residual visual impairment

## Abstract

**Background and Purpose:**

Cerebral venous thrombosis (CVT) is a special cerebrovascular disease that accounts for around 0.5%–1.0% of all strokes and often occurs in younger adults. Intracranial hypertension is the most frequent symptom of acute CVT due to venous occlusion. This study aimed to ascertain the risk factors for intracranial hypertension after CVT and to investigate whether intracranial hypertension at diagnosis may affect patient outcomes.

**Methods:**

We performed a retrospective cohort analysis of all patients treated for acute/subacute CVT at our department between 2018 and 2021. Logistic regression analysis was performed to identify potential risk factors associated with intracranial hypertension after CVT and clinical outcomes at the 6‐month follow‐up.

**Results:**

A total of 293 acute/subacute CVT survivors were eligible for inclusion, with 245 patients (83.60%) experiencing concomitant intracranial hypertension at diagnosis. In the multivariable regression analysis, hereditary thrombophilia (OR 2.210, 95% CI 1.148–4.254, *p* = 0.018) and thrombosis location of superior sagittal sinus (SSS) and right lateral sinus (LS) (OR 4.115, 95% CI 1.880–9.010, *p* = 0.000) were independently associated with intracranial hypertension. 83.67% of patients with intracranial hypertension after CVT had favorable functional outcomes (mRS score, 0–2), whereas they more often had residual visual impairment (15.51% vs. 4.17%, *p* = 0.036) at follow‐up. The risk factors for residual visual impairment were papilledema (OR 2.971, 95% CI 1.231–7.170, *p* = 0.015) and visual disturbances at diagnosis (OR 2.869, 95% CI 1.123–7.327, *p* = 0.028), thrombosis location (SSS and right LS [OR 10.811, 95% CI 4.208–27.773, *p* = 0.000]; SSS and left LS [OR 3.139, 95% CI 1.409–6.995, *p* = 0.005]), and CVT recurrence (OR 4.763, 95% CI 1.556–14.584, *p* = 0.006).

**Conclusions:**

Intracranial hypertension is the most common clinical symptom of acute CVT. At follow‐up, patients with intracranial hypertension after CVT were more prone to develop residual visual impairment.

## Graphical abstract

Cerebral venous thrombosis (CVT) is a special cerebrovascular disease that accounts for around 0.5%–1.0% of all strokes and often occurs in younger adults. Intracranial hypertension is the most frequent symptom of acute CVT due to impaired cerebral venous drainage and absorption of cerebrospinal fluid. Previous studies have addressed the relationship between CVT and intracranial hypertension, but the risk factors for intracranial hypertension after CVT and whether intracranial hypertension at diagnosis may affect patient outcomes are not completely understood. This retrospective cohort study collected 306 cases treated for acute/subacute CVT, investigated underlying risk factors for intracranial hypertension after CVT, and estimated the correlation between intracranial hypertension at diagnosis and clinical outcomes.

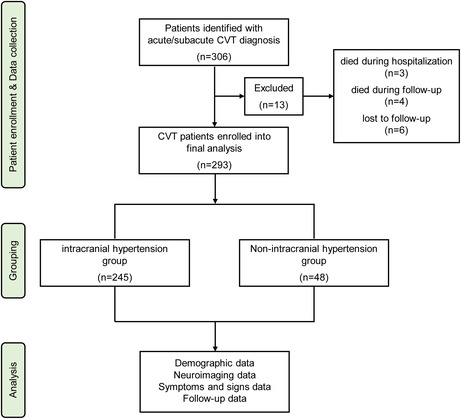

## 1 INTRODUCTION

Cerebral venous thrombosis (CVT) is a special cerebrovascular disease caused by thrombosis of the dural sinuses and/or intracranial veins,^1^, ^2^, ^3^ which accounts for around 0.5%–1.0% of all strokes.^4^, ^5^ This disease occurs more frequently in young and middle‐aged adults, with a sex ratio heavily skewed toward females.^1^, ^2^, ^3^ The clinical manifestation of CVT is highly variable, depending on the predominant site of venous occlusion. Approximately 60% of patients have CVT involving multiple dural venous sinuses, with the superior sagittal sinus (SSS) being the most frequently affected. CVT can also lead to intracranial hypertension due to impaired cerebral venous drainage and absorption of cerebrospinal fluid (CSF). Moreover, in isolated intracranial hypertension cases, patients may suffer from headaches (often accompanied by nausea), papilledema, visual field disturbances, and tinnitus.^1^

The risk factors and causes of CVT are complicated and diverse, involving sex‐specific factors such as oral contraceptive use, pregnancy, puerperium, hereditary thrombophilia, infections, and neurosurgical procedures.^6^, ^7^ The International Study on Cerebral Vein and Dural Sinus Thrombosis (ISCVT) found that up to 85% of adult patients have at least one risk factor^8^; some of the risk factors are also the leading triggers for intracranial hypertension, thus affecting the overall outcome and quality of life.^9^ Furthermore, patients with acute/subacute CVT may present predominantly with clinical features of intracranial hypertension, such as headache, blurred vision, transient visual obscuration, diplopia, and/or papilledema.^4^, ^10^ Previous studies have addressed the relationship between CVT and intracranial hypertension. This retrospective study aimed to investigate underlying risk factors for intracranial hypertension after CVT and to estimate the correlation between intracranial hypertension at diagnosis and clinical outcomes by analysis of a large cohort of CVT patients from our institution.

## 2 METHODS

### 2.1 Patient enrollment

In this retrospective cohort study, CVT patients were identified from a prospective stroke registry in Xuanwu Hospital, Capital Medical University. Patients experiencing first‐episode acute/subacute CVT were enrolled consecutively between January 2018 and June 2021. The diagnosis of CVT was based on various imaging modalities, including magnetic resonance imaging (MRI), magnetic resonance venography (MRV), computed tomography (CT), computed tomography venography (CTV), and/or digital subtraction angiography (DSA). Based on the interval from symptom onset to a confirmed diagnosis, the acute phase (0–7 days) and the subacute phase (8–15 days) were defined.^11^ Intracranial pressure was measured by lumbar puncture (LP) in the left lateral decubitus position, and a CSF opening pressure of 250 mmH_2_O or more was identified as intracranial hypertension.^12^, ^13^ We categorized visual outcomes into 2 conditions: residual visual impairment and non‐residual visual impairment. Visual impairment was defined as severe papilledema (Frisen grade ≥3), visual field defect or fading eyesight (more than 2 lines with Snellen visual chart).^14^, ^15^

All patients were strictly selected with the following inclusion criteria: (1) age ≥18 years; (2) acute/subacute CVT diagnosed by MRI + MRV, CT + CTV, or DSA; (3) neuro‐ophthalmological examination and color fundus photography performed at admission and 6‐month follow‐up; and (4) available valid information on functional outcomes and residual visual outcomes at 6‐month follow‐up. There were no restrictions regarding sex, and patients with malignancies were not included because the malignancies could be the direct cause of death or affect the outcomes. All participants signed informed consent forms before enrollment and data acquisition. This study was approved by the Ethics Committee of Xuanwu Hospital, Capital Medical University ([2020]098).

### 2.2 Data collection

The demographics, epidemiological data, radiological characteristics, treatments, and clinical outcomes of all patients were collected. Etiology and risk factors for CVT included sex‐specific factors (use of estrogen‐progesterone, puerperium, oral contraceptives, pregnancy), hereditary thrombophilia factors (protein C, S, or antithrombin III deficiency), acquired thrombophilia factors (antiphospholipid syndrome, nephrotic syndrome, hyperhomocysteinemia), and other risk factors (anemia, infections). The clinical symptoms and signs included intracranial hypertension, headache, papilledema, visual disturbances, epileptic seizure, motor deficits, aphasia, mental disorders, and Glasgow coma scale [GCS] score. Neuroimaging data comprised the location of the thrombus (SSS, bilateral lateral sinus [LS], and bilateral sigmoid sinus) and parenchymal changes (venous infarction and hemorrhage). Additionally, neuro‐ophthalmological examination and color fundus photography results at admission and follow‐up, treatments (heparin, endovascular treatment) in the acute phase, use of anticoagulant therapy at discharge, modified Rankin scale (mRS) score on admission and discharge, whether interventional thrombectomy was selected, and CVT recurrence during follow‐up were recorded. The patient data reporting followed the STROBE guideline.^16^

### 2.3 Treatment protocol

Patients with CVT were treated with a standard treatment protocol immediately after diagnosis according to the current guidelines.^17^ Each patient received subcutaneous low‐molecular‐weight heparin in adjusted doses for 10 to 14 days, followed by oral anticoagulants (warfarin or dabigatran, if warfarin was used, PT‐INR was maintained between 2.0 and 3.0) for 3–6 months or longer. The use of endovascular treatment (local thrombectomy/thrombolysis) was reserved for patients who are still progressing with adequate anticoagulant therapy. Short‐term therapy with mannitol or furosemide was administrated to patients with progressive visual loss or cerebral herniation due to intracranial hypertension, acetazolamide, decompressive craniectomy, ventriculoperitoneal shunt or hematoma evacuation, optic nerve sheath decompression was selected by physicians according to current guidelines.^17^

### 2.4 Follow‐up and clinical outcomes

Regular follow‐up was conducted 6 months after discharge. Follow‐up and outcome data were collected through clinical outpatient visits with a standardized questionnaire. Residual symptoms, such as headache, residual visual impairment, and current work status, were evaluated according to the proposed criteria.^9^, ^18^ Brain and ophthalmological characteristics were assessed using radiological imaging modalities. mRS score at the 6‐month follow‐up after discharge was used as the primary endpoint of efficacy. An mRS score ≤2 was defined as a relatively favorable outcome, whereas an mRS score ≥3 indicated a poor prognosis.

### 2.5 Statistics

Values of the measured parameters were checked for conformity to a normal distribution by means of the Kolmogorov–Smirnov test prior to statistical analysis. Continuous variables are expressed as the mean ± standard deviation (SD) or median with interquartile range (IQR), and categorical variables are expressed as percentages. Bivariate analysis with the *t* test or Mann–Whitney *U* test for continuous variables and the chi‐square test for categorical variables were used to identify the potential variables associated with intracranial hypertension at diagnosis (or residual visual impairment).

Bivariate logistic regression models were used to examine the associations between each of the potential risk factors and intracranial hypertension at diagnosis (or residual visual impairment) in all CVT survivors. First, with *p* ≤ 0.15 in the univariate analysis, etiologic and neuroimaging data were entered as dependent variables. Then, we checked collinearity among these potential predictors using the tolerance and variance inflation factors test. However, no significant collinearity was detected among any of the potential predictors. Next, all the retained predictors, together with age and sex as covariates, and intracranial hypertension at diagnosis (or residual visual impairment) as the dependent variable, were entered into the regression model. We calculated the odds ratios (ORs) and 95% confidence intervals (CIs) for the retained variables. A two‐sided *p* value ≤0.05 was considered significant. SPSS 22.0 for Windows (IBM Corp) was used to analyze all data.

## 3 RESULTS

**FIGURE 1:**
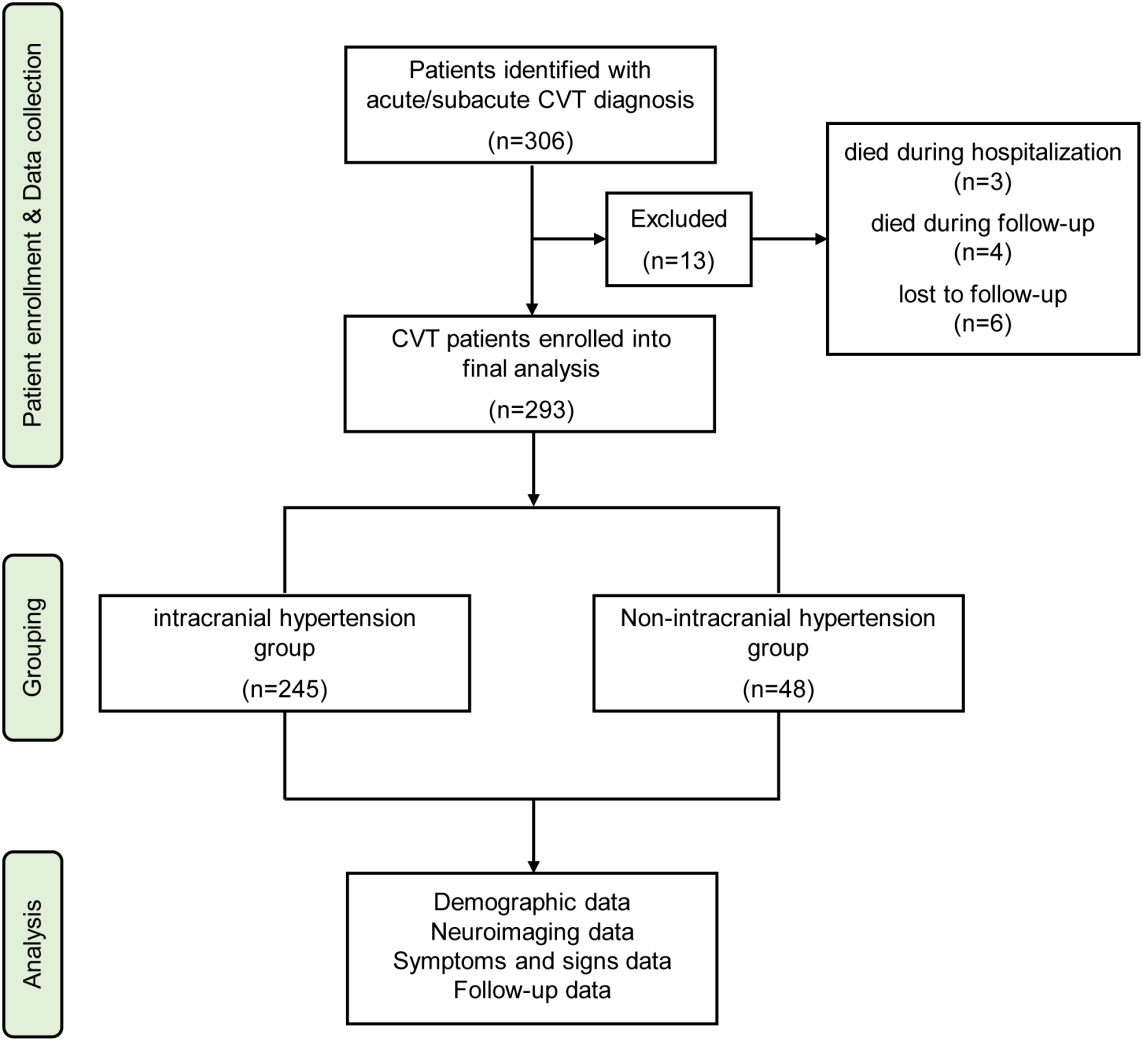
Flow chart of patient selection. CVT, cerebral venous thrombosis.

We identified 306 patients with diagnosed acute/subacute CVT. Of these, 3 patients (0.98%) died during hospitalization, 4 patients (1.31%) died during follow‐up, and 6 patients (1.96%) were lost to follow‐up. Thus, a total of 293 CVT survivors (median duration of follow‐up was 6 months [IQR 5.5–7.0]) were eligible for this study, including 245/293 (83.60%) intracranial hypertension patients (mean age was 35.51 years [IQR 18–81], and 60.82% were female) and 48/293 (16.40%) non‐intracranial hypertension patients (mean age was 38.88 years [IQR 18–87], and 62.50% were female). Figure 1 depicts the study flow chart.

### 3.1 Baseline characteristics and outcomes of all CVT survivors with and without intracranial hypertension

Among all survivors, univariate analysis revealed that the incidences of headache (234/245 [95.51%] vs. 41/48 [85.42%], *p* = 0.008), papilledema (109/245 [44.49%] vs. 11/48 [22.92%], *p* = 0.005), and visual disturbances (50/245 [20.41%] vs. 1/48 [2.08%], *p* = 0.002) were significantly higher in the intracranial hypertension group. Patients with intracranial hypertension more often had thrombosis of the SSS, right LS, right sigmoid sinus, and ≥2 sinuses occluded. At the 6‐month follow‐up, 84.98% of survivors achieved favorable functional outcomes (mRS, 0–2), but there were no significant differences between the intracranial hypertension and non‐intracranial hypertension groups (*p* = 0.156). Additionally, the incidence of residual visual impairment was significantly higher in the intracranial hypertension group (38/245 [15.51%] vs. 2/48 [4.17%], *p* = 0.036). The detailed baseline characteristics and follow‐up information for all CVT survivors are presented in Table 1.

**TABLE 1:** Baseline characteristics and functional outcomes of all CVT survivors with intracranial hypertension or without intracranial hypertension.

Variables	All cases (*n* = 293)	Non‐intracranial hypertension (*n* = 48)	Intracranial hypertension (*n* = 245)	*p*
Sex (female)	179 (61.09%)	30 (62.50%)	149 (60.82%)	0.827
Age (IQR), year	36.06 (18,87)	38.88 (18,87)	35.51 (18,81)	0.160
Risk factors				
Sex‐specific^a^	102 (34.81%)	8 (16.67%)	94 (38.37%)	0.004
Hereditary^b^	91 (31.06%)	22 (45.83%)	69 (28.16%)	0.016
Acquired^c^	107 (36.52%)	18 (37.50%)	89 (36.33%)	0.877
Symptoms and signs				
Headache	275 (93.86%)	41 (85.42%)	234 (95.51%)	0.008
Papilledema	120 (40.96%)	11 (22.92%)	109 (44.49%)	0.005
Visual disturbance	51 (17.41%)	1 (2.08%)	50 (20.41%)	0.002
Epileptic seizure	124 (42.32%)	25 (52.08%)	99 (40.41%)	0.134
Motor deficits	114 (38.91%)	21 (43.75%)	93 (37.96%)	0.452
Aphasia	62 (21.16%)	5 (10.42%)	57 (23.27%)	0.046
Mental disorders	74 (25.26%)	6 (12.50%)	68 (27.76%)	0.080
Coma (GCS <9)	81 (27.65%)	11 (22.92%)	70 (28.57%)	0.153
Neuroimaging				
Venous infarction	195 (66.55%)	35 (72.92%)	160 (65.31%)	0.307
Cerebral hemorrhage	111 (37.88%)	23 (47.92%)	88 (35.92%)	0.117
Location of thrombus				
Superior sagittal sinus	188 (64.16%)	22 (45.83%)	166 (67.76%)	0.004
Lateral sinus, right	146 (49.83%)	14 (29.17%)	132 (53.88%)	0.002
Lateral sinus, left	126 (43.00%)	16 (33.33%)	110 (44.90%)	0.139
Sigmoid sinus, right	126 (43.00%)	13 (27.08%)	113 (46.12%)	0.015
Sigmoid sinus, left	118 (40.27%)	15 (31.25%)	103 (42.04%)	0.163
SSS & (LS, right)	114 (38.91%)	8 (16.67%)	106 (43.27%)	0.001
SSS & (LS, left)	86 (29.35%)	10 (20.83%)	76 (31.02%)	0.156
≥2 sinuses occluded	156 (53.24%)	17 (35.42%)	139 (56.73%)	0.007
Follow‐up and functional outcome				
mRS, 0–2	249 (84.98%)	44 (91.67%)	205 (83.67%)	0.156
Neurologic defects	64 (21.84%)	8 (16.67%)	56 (22.86%)	0.343
Residual visual impairment	40 (13.65%)	2 (4.17%)	38 (15.51%)	0.036
CVT recurrence	25 (8.53%)	3 (6.25%)	22 (8.98%)	0.536

Abbreviations: CVT, cerebral venous thrombosis; GCS, Glasgow Coma Scale; IQR, interquartile range; LS, lateral sinus; mRS, modified Rankin Scale; SSS, Superior sagittal sinus.

^a^ Oral contraceptives, pregnancy/puerperium, and/or hormone replacement therapy.

^b^ Protein C, protein S, and/or antithrombin III deficiency.

^c^ Antiphospholipid antibodies, anticardiolipin antibodies, nephrotic syndrome, and/or hyperhomocysteinemia.

### 3.2 Risk factors for intracranial hypertension in all CVT survivors

Bivariate logistic analysis was performed to identify independent risk factors for intracranial hypertension in all CVT survivors. However, no significant collinearity was detected among any of the potential predictors. Together with age and sex (females), risk factors (sex‐specific factors and hereditary thrombophilia), thrombosis location (SSS, right LS, left LS, right sigmoid sinus, SSS and right LS, ≥2 sinuses occluded), headache, papilledema, visual disturbance, epileptic seizure, aphasia, mental disorders, hemorrhage, and residual visual impairment were entered as dependent variables into the regression model.

Finally, the logistic model revealed the statistically significant factors (χ^2^ = 33.815, *p* < 0.001). Among the independent variables included in the model, age, sex‐specific factors, hereditary thrombophilia, and thrombosis of SSS and right LS were statistically significant. We calculated the odds ratios (ORs) and 95% confidence intervals (CIs) of the retained variables. In the multivariable logistic regression model, age (OR 0.979, 95% CI 0.960–0.999, *p* = 0.036), sex‐specific factors (OR 0.262, 95% CI 0.113–0.605, *p* = 0.002), hereditary thrombophilia (OR 2.210, 95% CI 1.148–4.254, *p* = 0.018), and thrombosis of SSS and right LS (OR 4.115, 95% CI 1.880–9.010, *p* = 0.000) were independently associated with intracranial hypertension after CVT. The details are displayed in Table 2.

**TABLE 2:** Risk factors for intracranial hypertension in all CVT survivors.

Potential risk factors	OR	95% CI	*p*
Age	0.979	0.960–0.999	0.036
Sex‐specific^a^	0.262	0.113‐0.605	0.002
Hereditary^b^	2.210	1.148–4.254	0.018
Thrombosis of SSS and right LS	4.115	1.880–9.010	0.000

Abbreviations: CI, confidence interval; CVT, cerebral venous thrombosis; LS, Lateral sinus; OR, odds ratio; SSS, Superior sagittal sinus.

^a^ Oral contraceptives, pregnancy/puerperium, and/or hormone replacement therapy.

^b^ Protein C, protein S, and/or antithrombin III deficiency.

### 3.3 Baseline characteristics and outcomes of patients with and without residual visual impairment

Since most CVT patients with severe intracranial hypertension usually experienced decreased visual function, 293 survivors in this study were classified into residual visual impairment (*n* = 40) and non‐residual visual impairment (*n* = 253) groups. Overall, univariate analysis revealed that intracranial hypertension (38/40 [95.00%] vs. 207/253 [81.82%], *p* = 0.036), papilledema (28/40 [70.00%] vs. 92/253 [36.36%], *p* = 0.000), and visual disturbances (15/40 [37.50%] vs. 36/253 [14.23%], *p* = 0.000) significantly differed between the groups. Moreover, patients with residual visual impairment appeared to have a higher likelihood of a suspected thrombosis location, including SSS, right LS, and ≥2 sinuses occluded (Table S1).

### 3.4 Risk factors for patients with residual visual impairment

Using multivariate logistic regression analysis, papilledema at diagnosis (OR 2.971, 95% CI 1.231–7.170, *p* = 0.015), visual disturbances at diagnosis (OR 2.869, 95% CI 1.123–7.327, *p* = 0.028), thrombosis of SSS and right LS (OR 10.811, 95% CI 4.208–27.773, *p* = 0.000), thrombosis of SSS and left LS (OR 3.139, 95% CI 1.409–6.995, *p* = 0.005), and CVT recurrence (OR 4.763, 95% CI 1.556–14.584, *p* = 0.006) were independently associated with residual visual impairment. The details are displayed in Table 3.

**TABLE 3:** Risk factors for patients with residual visual impairment.

Potential risk factors	OR	95% CI	*p*
Papilledema	2.971	1.231–7.170	0.015
Visual disturbance	2.869	1.123–7.327	0.028
Thrombosis of SSS and right LS	10.811	4.208–27.773	0.000
Thrombosis of SSS and left LS	3.139	1.409–6.995	0.005
CVT recurrence	4.763	1.556–14.584	0.006

Abbreviations: CI, confidence interval; CVT, cerebral venous thrombosis; LS, Lateral sinus; OR, odds ratio; SSS, Superior sagittal sinus.

## 4 DISCUSSION

In the present study, 83.60% of CVT survivors presented primarily with symptoms of intracranial hypertension and achieved a favorable functional outcome (83.67%) at follow‐up. CVT has a wide spectrum of clinical presentation syndromes and falls broadly into four categories^4^, ^19^: symptoms and signs of raised intracranial pressure (ICP), focal neurological deficits (often in combination with seizures), diffuse encephalopathy, and cavernous sinus thrombosis. To the best of our knowledge, intracranial hypertension is the most prominent manifestation after CVT, characterized by increased ICP. In the ISCVT prospective cohort, LP with measurement of the opening pressure was performed in 224 patients (35.9%), and a raised ICP was found in 83.3% of the 127 patients in whom it was recorded.^20^ Therefore, intracranial hypertension in patients with acute/subacute CVT is quite common.

To date, only a few studies have analyzed differences in CVT patients with and without intracranial hypertension. CVT has multiple risk factors and associated conditions, yet the underlying etiological factors for intracranial hypertension after CVT remain uncertain. In our study, age, female sex, and hereditary thrombophilia were independently associated with intracranial hypertension, which is similar to the clinical characteristics of CVT. As reported, a majority of patients have multiple sites of thrombosis, but the distribution might vary.^1^, ^4^, ^8^ The SSS, LS, and sigmoid sinus are the most common sites. In a retrospective study of 160 patients diagnosed with CVT, Sassi et al. found that multiple venous sinuses (114 cases, 71.2%) were involved, and the SSS was the most frequently involved (65%), followed by the transverse sinuses (60.5%).^21^ In the ISCVT, the most common thrombosis sites were the sagittal sinus (62%) and transverse sinuses (left: 44.7%, right: 41.2%); simultaneously, 18% of patients exhibited straight sinus involvement.^8^ Our patients who developed intracranial hypertension were also prone to having multiple venous sinuses being involved at diagnosis. The SSS was involved in 166 (67.76%) patients, the right LS was involved in 132 (53.88%) patients, and the right sigmoid sinus was involved in 113 (46.12%) patients. In addition, the thrombosis location of SSS and right LS is a significant risk factor for intracranial hypertension after CVT. These findings indicate that intracranial hypertension may be secondary to venous sinus thrombosis, owing to impaired venous return in patients with CVT.

The cause of intracranial hypertension after CVT is unknown but probably involves obstruction of the cerebral venous outflow due to impaired cerebral venous drainage and absorption of CSF.^22^, ^23^ Once a thrombus is formed in the cerebral veins, its expansion can occlude large draining venous sinuses, sequentially creating physiological backpressure in the venous system. First, CVT can directly induce increased pressure in the venules and capillaries and reduced cerebral perfusion and venous sinus flow, resulting in slowing of brain circulation. In addition, CVT can block CSF absorption through the arachnoid villi, which is typically associated with SSS obstruction. Such dysfunction results in diminished drainage of the CSF and subsequently intracranial hypertension. Additionally, persistent sinus occlusion or persistently elevated ICP after CVT contributes to occult or accumulated brain tissue damage, sometimes leading to cerebral edema, local ischemia, and often intracerebral hemorrhage.^1^, ^24^ Our findings indicated that prompt diagnosis based on intracranial hypertension‐related symptoms and signs and early treatment, such as sinus recanalization, of patients with intracranial hypertension may significantly improve the outcome of CVT, especially in extremely severe cases.

CVT can trigger a constellation of neuro‐ophthalmological symptoms, such as papilledema, vision loss, and visual field defects. Papilledema is optic nerve head edema secondary to raised ICP. As a common manifestation of CVT, papilledema was observed in 28%–67.5% of patients,^8^, ^25^, ^26^ and visual disturbances were found, to varying degrees, in 13.2%–52.5% of patients.^8^, ^26^ A retrospective study conducted by Eliseeva et al. found that 30.6% of papilledema cases were in acute CVT patients, and 22.4% were in subacute CVT patients.^26^ Our results showed that 44.49% of patients with intracranial hypertension regard papilledema as an initial symptom. The frequency of residual visual impairment in our series (13.65% in survivors) was similar to that in the ISCVT (13% among survivors),^8^ probably due to the similar definition of residual visual impairment. Another explanation for the similar frequencies could be the large sample size.

The possible pathophysiologic mechanism causing residual visual impairment in CVT involves elevated ICP due to venous thrombosis. In our study, logistics analysis indicated that the thrombosis location of SSS and right LS as well as SSS and left LS were independently associated with residual visual impairment. Visual disturbances occurred in 20.41% of the CVT patients with intracranial hypertension at diagnosis, while residual visual impairment at the 6‐month follow‐up had no significant relationship with intracranial hypertension after CVT. The reason may be because the properties of residual visual impairment in CVT are diverse, and residual visual impairment after CVT can also be caused by focal lesions such as cerebral infarction and hemorrhage. Additionally, our results showed that CVT recurrence was one of the risk factors for poor visual outcomes. Therefore, patients with CVT require regular monitoring of visual function, with both central acuity and visual field analysis, to prevent/reduce irreversible visual outcomes.

There are some limitations to our study. First, this retrospective study included a relatively small number of cases, and this was a single‐center study with only Chinese patients. This may cause selection bias and limit the generalizability of the results. Moreover, we cannot absolutely exclude that the absence of an association between residual visual impairment and intracranial hypertension after CVT is due to the limited sample size. A prospective, multicenter, large‐sample trial is needed to verify our findings. Second, there was a lack of baseline characteristics, such as height and weight at admission, that could have underpowered the study and biased the results because obesity is also a risk factor for poor visual outcomes in CVT.^27^ Future studies should measure and record patients' height and weight in their medical history. Understanding the roles of multiple risk factors for residual visual impairment after CVT is essential to guiding treatment decisions in the future. Third, the follow‐up period was 6 months, and poor visual outcomes may have occurred later, so longer follow‐up times are still necessary.

The strength of this study lies in the low missed follow‐up rate and uniform treatment approach, thus minimizing confusion regarding the outcomes and allowing for a sufficiently accurate evaluation of risk factors for intracranial hypertension or residual visual impairment after CVT.

## 5 CONCLUSIONS

Intracranial hypertension frequently occurs during the acute phase of CVT. Age, sex‐specific factors, hereditary thrombophilia, and thrombosis location of SSS and right LS increased the risk for intracranial hypertension after CVT. At follow‐up, patients with intracranial hypertension after CVT were more prone to develop residual visual impairment. Papilledema or visual disturbances at diagnosis, thrombosis location (SSS and right LS or SSS and left LS), and CVT recurrence were independently associated with residual visual impairment.

## AUTHOR CONTRIBUTION

HW, HJ, CZ, and XJ researched literature and conceived the study. HW, HJ, and CZ were involved in protocol development. HW, HJ, YZ, LL, CZ, and XJ were involved in gaining ethical approval, patient recruitment, and samples collection. HW, HJ, and CZ were involved in sample analysis and interpretation of the results. HW and CZ wrote the first draft of the manuscript. All authors reviewed and edited the manuscript and approved the final version of the manuscript. XJ is responsible for the overall content as the guarantor.

## FUNDING INFORMATION

This study was supported by grants from National Natural Science Foundation of China (82271311) and the Pharmaceutical Collaboration Project of Beijing Science and Technology Commission (Z181100001918026).

## CONFLICT OF INTEREST STATEMENT

The authors declare that they have no conflicts of interest.

## Supporting information


Data S1.

## Data Availability

Anonymized raw data are available upon request from the corresponding author.
