# Supplementary material for: Intracranial hypertension after cerebral venous thrombosis—Risk factors and outcomes
Source: CNS Neurosci Ther. 2023 Mar 28;29(9):2540–7. doi: 10.1111/cns.14194 (PMC10401171; doi:10.1111/cns.14194)
Supplement: Supplementary file 1 — Data S1. [file CNS-29-2540-s001.docx]

**SUPPLEMENTARY DATA**

**Intracranial hypertension after** **cerebral venous thrombosis - risk factors and outcomes**

Huimin Wei^1,2†^, Huimin Jiang^2†^, Yifan Zhou^2^, Lu Liu^3^, Chen Zhou^2^^*^, Xunming Ji^1,2,4*^

**Supplementary Table 1.** ***Baseline characteristics and functional outcomes in patients with residual visual impairment or without residual visual impairment***

| **Variables** | **All cases**  **(n=293)** | **Non-residual visual impairment (n=253)** | **Residual visual impairment (n=40)** | ***P*** |
| --- | --- | --- | --- | --- |
| Sex (female) | 179 (61.09%) | 154 (60.87%) | 25 (62.50%) | 0.844 |
| Age (IQR), year | 36.06 (18,87) | 35.66 (18,87) | 38.63 (18,74) | 0.251 |
| Risk factors | | | | |
| Sex-specific^a^ | 102 (34.81%) | 93 (36.76%) | 9 (22.50%) | 0.079 |
| Hereditary^b^ | 91 (31.06%) | 80 (31.62%) | 11 (27.50%) | 0.601 |
| Acquired^c^ | 107 (36.52%) | 91 (35.97%) | 16 (40.00%) | 0.623 |
| Symptoms and signs | | | | |
| Intracranial hypertension | 245 (83.62%) | 207 (81.82%) | 38 (95.00%) | 0.036 |
| Headache | 275 (93.86%) | 237 (93.68%) | 38 (95.00%) | 0.746 |
| Papilledema | 120 (40.96%) | 92 (36.36%) | 28 (70.00%) | 0.000 |
| Visual disturbance | 51 (17.41%) | 36 (14.23%) | 15 (37.50%) | 0.000 |
| Epileptic seizure | 124 (42.32%) | 113 (44.66%) | 11 (27.50%) | 0.041 |
| Motor deficits | 114 (38.91%) | 95 (37.55%) | 19 (47.50%) | 0.230 |
| Aphasia | 62 (21.16%) | 50 (19.76%) | 12 (30.00%) | 0.141 |
| Mental disorders | 74 (25.26%) | 60 (23.72%) | 14 (35.00%) | 0.228 |
| Coma (GCS < 9) | 81 (27.65%) | 71 (28.06%) | 10 (25.00%) | 0.768 |
| Neuroimaging | | | | |
| Venous infarction | 195 (66.55%) | 170 (67.19%) | 25 (62.50%) | 0.559 |
| Cerebral hemorrhage | 111 (37.88%) | 97 (38.34%) | 14 (35.00%) | 0.686 |
| Location of thrombus | | | | |
| Superior sagittal sinus | 188 (64.16%) | 152 (60.08%) | 36 (90.00%) | 0.000 |
| Lateral sinus, right | 146 (49.83%) | 111 (43.87%) | 35 (87.50%) | 0.000 |
| Lateral sinus, left | 126 (43.00%) | 105 (41.50%) | 21 (52.50%) | 0.192 |
| Sigmoid sinus, right | 126 (43.00%) | 105 (41.50%) | 21 (52.50%) | 0.192 |
| Sigmoid sinus, left | 118 (40.27%) | 101 (39.92%) | 17 (42.50%) | 0.757 |
| SSS & (LS, right) | 114 (38.91%) | 81 (32.02%) | 33 (82.50%) | 0.000 |
| SSS & (LS, left) | 86 (29.35%) | 65 (25.69%) | 21 (52.50%) | 0.001 |
| ≥2 sinuses occluded | 156 (53.24%) | 125 (49.41%) | 31 (77.50%) | 0.001 |
| Follow-up and functional outcome | | | | |
| mRS,0-2 | 249 (84.98%) | 217 (85.77%) | 32 (80.00%) | 0.342 |
| Neurologic defects | 64 (21.84%) | 50 (19.76%) | 14 (35.00%) | 0.030 |
| CVT recurrence | 25 (8.53%) | 16 (6.32%) | 9 (22.50%) | 0.001 |

Abbreviations: IQR, interquartile range; GCS, Glasgow Coma Scal; mRS, modified Rankin Scale; SSS, Superior sagittal sinus; LS, Lateral sinus; CVT: cerebral venous thrombosis.

a Oral contraceptives, pregnancy/puerperium, and/or hormone replacement therapy.

b Protein C, protein S, and/or antithrombin III deficiency.

c Antiphospholipid antibodies, anticardiolipin antibodies, nephrotic syndrome, and/or hyperhomocysteinemia.
